# The extent of coercion in psychiatric emergency room based in Polish general hospital.

**DOI:** 10.1192/j.eurpsy.2023.1843

**Published:** 2023-07-19

**Authors:** M. Z. Zarzycki, U. Zaniewska-Chłopik, M. Załuska

**Affiliations:** 1 Clinical Department of Psychiatry; 2 Centre of Mental Health, Bielanski Hospital, Warsaw, Poland

## Abstract

**Introduction:**

Coercion in psychiatric wards may improve the safety of patients and surroundings, on the other hand, its use affects compliance and satisfaction with treatment. In Poland the coercive measures are strictly regulated by The Mental Health Act (1994). Most of published studies refers to the coercion only during hospitalisation.

**Objectives:**

Assessment of the extent of coercive measures in psychiatric emergency room and evaluation of the relationships between the use of direct coercion and selected demographic-clinical factors.

**Methods:**

This study was conducted at the Bielanski Hospital in Warsaw on all the patients admitted to the psychiatric ward over one year. The extent of coercion in the psychiatric emergency room, demographic and clinical data were collected. Patients were assessed in Brief Psychiatric Rating Scale (BPRS) prior to admission. Patients’ sociodemographic and clinical factors were tested in a multivariate logistic regression model.

**Results:**

In the study 318 patients were included. Coercion of some form in the psychiatric emergency room was used in 29% of cases: admission without consent in 22% of cases and direct coercion (holding, forced medication, mechanical restraint) in 7%. Use of direct coercion in the psychiatric emergency room was associated with BPRS scoring: positively with severity of disorientation symptoms and negatively with severity of depression symptoms. Suicide attempts in the past were discovered to reduce the risk of being a subject of coercive measures. We found no demographic data associated in any way with coercion use.

**Conclusions:**

Coercion in psychiatric emergency room was related to patients’ mental state and their past medical history. There is no evidence of coercive measures misuse towards any demographic group.

**Disclosure of Interest:**

None Declared

